# Patient-Specific Vascular Anatomy for Bedside Safety: A Targeted Narrative Review and the VASC-N Framework

**DOI:** 10.3390/nursrep16090333

**Published:** 2026-09-10

**Authors:** Juan Alberto Sanchis-Gimeno, Andreea-Bianca Zuld, Jose E. León-Rojas, Juan José Valenzuela-Fuenzalida, Alejandro Bruna-Mejías, Gustavo Oyanedel-Amaro, Mathias Orellana-Donoso

**Affiliations:** 1 GIAVAL Research Group, Department of Human Anatomy and Embryology, Faculty of Medicine and Dentistry, University of Valencia, 46010 Valencia, Spain; juan.sanchis@uv.es (J.A.S.-G.); ab10zuld@gmail.com (A.-B.Z.); 2 Cerebro, Emoción y Conducta (CEC) Research Group, Escuela de Medicina, Universidad de Las Américas (UDLA), 170124 Quito, Ecuador; 3 Departamento de Ciencias Químicas y Biológicas, Facultad de Ciencias de la Salud, Universidad Bernardo O’Higgins, 8320000 Santiago, Chile; juan.kine.2015@gmail.com; 4 Departamento de Morfología, Facultad de Medicina, Universidad Andrés Bello, 8370035 Santiago, Chile; alejandro.bruna@unab.cl (A.B.-M.); mathor94@gmail.com (M.O.-D.); 5 Departamento de Ciencias y Geografía, Facultad de Ciencias Naturales y Exactas, Universidad de Playa Ancha, 2360004 Valparaíso, Chile; 6 Faculty of Health and Social Sciences, University of the Americas, Santiago 8370040, Chile; g.oyanedelamaro@gmail.com; 7 Escuela de Medicina, Universidad Finis Terrae, Santiago 7501015, Chile

**Keywords:** anatomical variation, vascular anatomy, health professions education, nursing education, vascular access, ultrasound, patient safety, simulation, competency-based education, person-centred care

## Abstract

Canonical vascular diagrams do not capture patient-specific differences in vessel identity, course, depth, calibre, branching, adjacency, dynamic behaviour, or post-procedural route. This targeted narrative review with purposive publication selection had two objectives: to synthesise vascular anatomical evidence relevant to nursing and health professions practice and education, and to use that synthesis to propose an action-oriented conceptual framework. PubMed/MEDLINE and the Web of Science Core Collection were searched from inception to 2 August 2026. After exact DOI and normalised-title deduplication, 1683 unique records underwent title/abstract inspection and keyword-assisted prioritisation; 56 information-rich human anatomical, ultrasonographic, clinical, educational, and sentinel case publications were purposively retained for full-text evidence charting and narrative synthesis. The synthesis identified six interpretive anatomy-to-risk mechanisms: possible vessel misidentification, unsafe neurovascular adjacency, device–vessel mismatch, dynamic positional change, misinterpretation of anomalous central routes, and loss of patient-specific anatomical knowledge between encounters. These categories are qualitative synthesis outputs, not frequency estimates or causal effects. The literature informed VASC-N—Verify; Assess; Scan and distinguish; Choose, adapt, and confirm; and Note, notify, and nurture learning—as an author-developed conceptual framework of proposed observable behaviours. A preliminary Nursing Actionability Test and minimum reporting checklist were also developed. Patient-specific vascular anatomy becomes educationally and clinically relevant when recognition may change a decision, support proportionate verification or escalation, preserve future access, or strengthen assessment. VASC-N and the accompanying tools have not been validated; prospective content-validity, feasibility, reliability, educational-transfer, and clinical-outcome studies are required.

## 1. Introduction

Blood sampling, peripheral intravenous cannulation, arterial puncture, vascular-device surveillance, peripherally inserted central catheter (PICC) and midline pathways, assistance with central access, and haemodialysis-access care are common components of nursing and interprofessional practice. Anatomy is more readily valued when linked to authentic clinical problems, while competency-based education requires integration of knowledge, judgement, technical execution, communication, and escalation [1,2,3,4,5]. Simulation and deliberate practice can improve procedural performance, and ultrasound can connect living anatomy with clinical reasoning, although curricula and outcome measures remain heterogeneous [6,7,8,9,10,11]. Any proposed assessment framework therefore requires explicit validity evidence for its intended interpretations and uses [12].

Vascular procedures are often introduced through canonical diagrams and landmark relationships. Such representations are useful starting models but cannot represent every patient or every procedural condition. At the bedside, vessel position, depth, calibre, patency, overlap, and accessibility may differ from the expected pattern and may change with positioning, probe pressure, respiration, previous interventions, or disease. Accordingly, procedural success should not be framed as a psychomotor task alone; it also requires calibrated anatomical judgement and an appropriate response when observed findings conflict with the canonical model.

In this review, patient-specific vascular anatomy is the overarching concept used for the vascular configuration relevant to a particular patient and encounter. Four non-equivalent categories are distinguished: (1) discrete congenital variants in origin, number, branching, persistence, absence, or course; (2) continuous anatomical variability in vessel diameter, depth, tortuosity, mobility, or distance from adjacent structures; (3) dynamic anatomical change related to limb or head position, probe pressure, respiration, intrathoracic pressure, or venous filling; and (4) acquired or remodelled anatomy after thrombosis, previous catheters, trauma, surgery, lymph-node procedures, or arteriovenous access creation. Only the first category necessarily corresponds to a classical named anatomical variant. The broader term prevents position-dependent or acquired states from being mislabelled as congenital anomalies while retaining their procedural relevance.

These distinctions have practical consequences. In the cubital fossa, variable superficial veins and nerve–vein crossings may make a familiar puncture site unsuitable [13,14,15,16]. Superficial upper-limb arteries may resemble veins and alter pulse location or catheter trajectory [17,18,19,20]. Internal jugular vein position and carotid overlap vary by level and head position [21,22,23,24], while upper-arm venous patterns can affect PICC, midline, or haemodialysis planning [25,26,27,28]. Femoral and axillary relationships and the small set of nursing-oriented reports further illustrate vessel overlap, unexpected catheter routes, and living-anatomy education [29,30,31,32,33,34]. Such evidence does not establish that anatomy alone causes a complication; it identifies possible mechanisms, uncertainty signals, and circumstances in which additional verification or a different plan may be justified.

The evidence is dispersed across anatomy, radiology, anaesthesia, nephrology, vascular-surgery, emergency-medicine, vascular-access, nursing, and education journals. Descriptive studies often establish morphology or prevalence, whereas procedural and educational studies may not make the anatomical mechanism explicit. The present work therefore has two linked but methodologically distinct objectives: first, to purposively synthesise evidence on patient-specific vascular anatomy that is relevant to nursing and health professions practice and education; and second, to use that synthesis to propose VASC-N, an action-oriented conceptual framework, together with preliminary publication and reporting tools. These outputs are proposals for validation, not empirically established competency instruments or safety interventions.

## 2. Materials and Methods

### 2.1. Review Design and Reporting Approach

This work was designed as a structured targeted narrative review with purposive publication selection and conceptual framework development. The source literature includes cadaveric anatomy, living ultrasonographic mapping, observational cohorts, systematic reviews, professional recommendations, educational studies, and sentinel case reports. These designs answer different questions, use incompatible units and endpoints, and cannot be meaningfully pooled into one effect or prevalence estimate. A narrative approach was therefore chosen to integrate anatomical constructs, plausible mechanisms, recognition cues, practice adaptations, educational implications, and research gaps across evidence types. The manuscript was structured with reference to the six SANRA domains [35].

The review was not registered as, and should not be interpreted as, a systematic or scoping review. It did not aim to exhaustively catalogue every vascular variant, estimate a single effect, or map the full field using duplicate independent screening. Instead, reproducible database searches created an auditable source pool from which a clinically information-rich set was purposively selected for interpretive synthesis and framework generation. This design permits cross-design conceptual integration but increases susceptibility to selection and interpretation bias; the resulting sample and framework are hypothesis-generating, non-comprehensive, and require prospective validation.

### 2.2. Information Sources and Search Strategies

PubMed/MEDLINE and the Web of Science Core Collection were searched from database inception to 2 August 2026, which remained the final evidence cut-off; the searches were not rerun after that date. PubMed/MEDLINE was selected for biomedical, clinical, nursing, and health-professions indexing, while the Web of Science Core Collection broadened multidisciplinary coverage and citation-linked discovery. This deliberately bounded two-database strategy was feasible for a targeted narrative review but was not intended to maximise nursing- or education-specific sensitivity. CINAHL, ERIC, Embase, and Scopus were not searched; their omission may have excluded relevant nursing, allied-health, education, or international records and is treated as a limitation. The strategies combined terms for anatomical variation, blood vessels, and nursing/procedural contexts. This focus increased clinical relevance but may have missed anatomically relevant papers whose titles and abstracts did not use nursing or procedural terminology. Complete platform-specific strategies are reproduced in Appendix A.

The final PubMed/MEDLINE export contained 656 records. The Web of Science Core Collection export was supplied in two consecutive RIS batches containing 1000 and 431 records, respectively, and these batches were treated as one 1431-record search result.

### 2.3. Record Management and Deduplication

Bibliographic records were parsed from the native PubMed .nbib file and the Web of Science RIS files. Metadata fields were normalised for title, authors, journal, year, DOI, PMID, abstract, keywords, and database provenance. Exact DOI matching was applied first; records without a DOI were compared by a normalised title string after case-folding, diacritic removal, and punctuation/spacing normalisation. No fuzzy match was used to remove records. This exact-match process removed 404 duplicates from 2087 database records and left 1683 unique records: 401 indexed in both databases, 1028 unique to Web of Science, and 254 unique to PubMed.

### 2.4. Eligibility and Purposive Selection

Stage 1 considered topical eligibility. Publications were eligible for prioritisation when they involved living humans or human anatomical material and addressed a macroscopic vascular variant, patient-specific vessel relationship, dynamic anatomical behaviour, or acquired/remodelled vascular anatomy with a plausible implication for a nursing-performed, nurse-managed, nurse-monitored, nurse-assisted, or nursing-taught procedure. Stage 2 applied purposive information-rich selection. A publication was prioritised when it contributed at least one clearly identifiable element: an anatomical construct; a denominator or distribution; an empirically observed association or plausible mechanism of misidentification, unsafe adjacency, failed access, malposition, thrombosis, infiltration, dysfunction, pain, or access loss; a bedside/imaging cue; a feasible adaptation, stop, confirmation, or escalation criterion; or a patient-, device-, clinician-, learner-, or service-level outcome.

Animal studies, foetal-only studies, molecular or genetic variants, nonvascular anatomy, and publications confined to operative or endovascular technique without a transferable nursing or educational implication were not retained for detailed charting. Cosmetic and specialty injection records were not retained unless they addressed a generalisable vascular-safety mechanism. Injection terms were intentionally included, but the retrieved evidence did not form a coherent body on routine nurse-administered intramuscular or subcutaneous injections; this absence was treated as a research gap rather than filled with unrelated literature.

**Figure 1:**
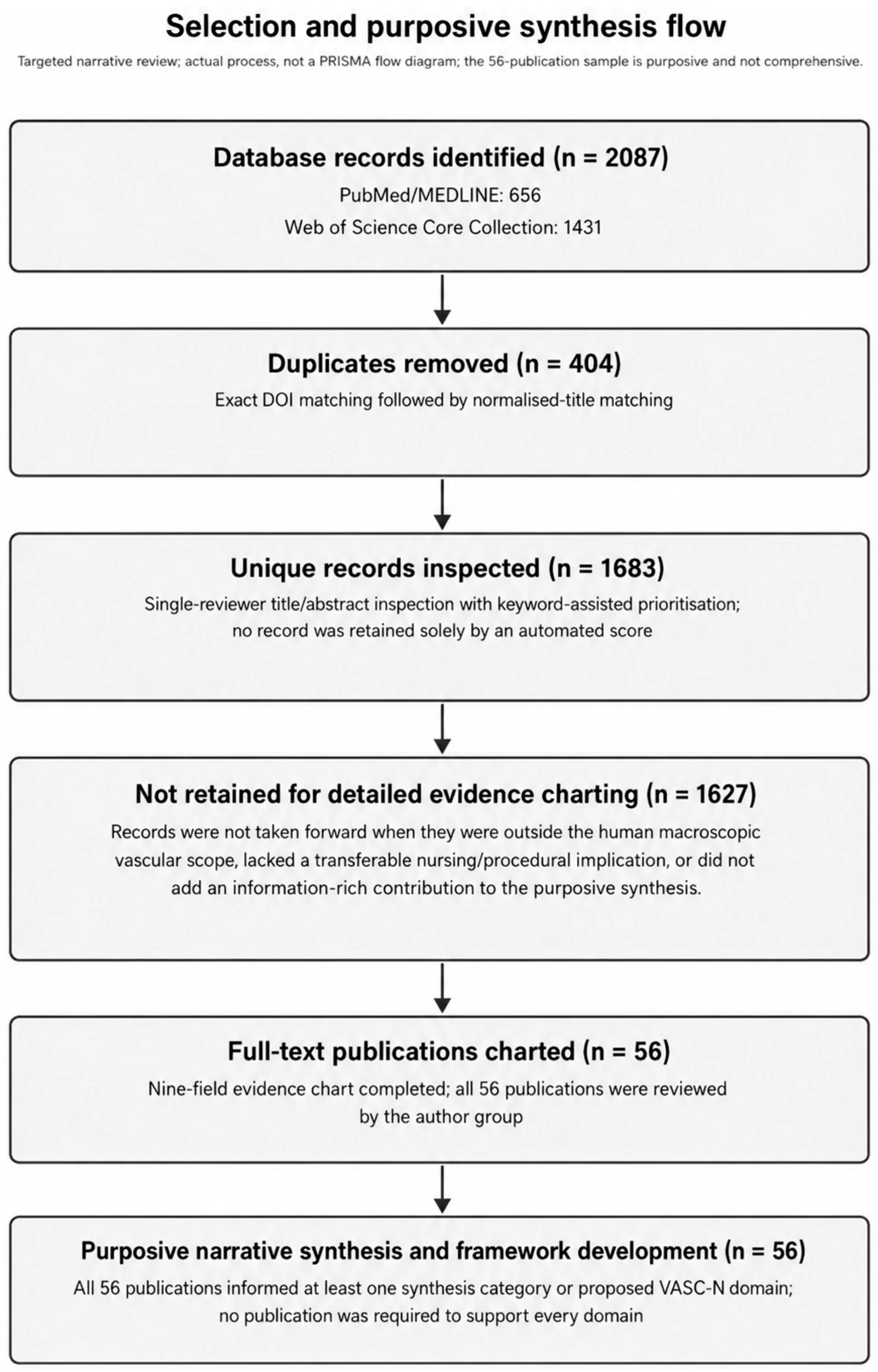
Actual selection and purposive synthesis flow. The diagram reports single-reviewer title/abstract inspection with keyword-assisted prioritisation, subsequent author-group review of the 56 charted publications, and the absence of a prospectively logged systematic full-text exclusion stage. It is not a PRISMA flow diagram, and the 56-publication sample should not be interpreted as all eligible studies or as a comprehensive representation of the literature.

All 1683 unique records entered title/abstract inspection and keyword-assisted prioritisation by one reviewer. A total of 1627 records were not retained for detailed evidence charting because they were outside the defined scope, lacked a transferable nursing/procedural implication, or did not add a distinct information-rich contribution to the purposive synthesis. Fifty-six publications were taken forward to full-text charting, and all 56 entered the narrative synthesis. Because this was not a systematic review, a separate prospectively logged full-text exclusion stage and reason-by-reason exclusion count were not maintained; retrospective counts were not created. Figure 1 reports the actual selection flow. To reduce subjectivity, selection was guided by explicit criteria, no record was retained solely because of an automated relevance score, every retained publication was charted in a standardised form, and the complete 56-publication chart was critically reviewed by the full author group. Differences concerning interpretation, evidence directness, or framework relevance were resolved through discussion. This author-group review did not constitute duplicate independent screening. The 56-publication set therefore constitutes a purposively selected, information-rich analytical sample and should not be interpreted as all potentially eligible studies or as a comprehensive or representative account of the literature.

### 2.5. Data Charting and Narrative Synthesis

For each publication selected for detailed synthesis, evidence was charted across study design; sample size and population or specimen source; anatomical territory; exact anatomical construct; ascertainment method; nursing procedure or management context; main result; mechanism or uncertainty signal; feasible adaptation, confirmation, or escalation; relevant outcome; methodological limitations; and proposed VASC-N contribution. Evidence was then synthesised by clinical task: superficial venipuncture, peripheral and upper-arm access, arterial identification and cannulation, internal jugular and central access, axillary and femoral access, anomalous thoracic venous routes, haemodialysis access, and education.

No single risk-of-bias instrument was applied across all source types because the purposively selected set included incompatible designs and the review did not estimate a pooled causal effect. Interpretation was weighted according to study design, denominator clarity, living versus cadaveric ascertainment, reproducibility of the anatomical method, directness to nursing action, and whether a clinical implication was directly measured, statistically associated, recommended by a professional source, biologically plausible, or inferred by the review authors. For interpretive clarity, three evidence-status categories were used: direct finding, meaning an anatomical feature, association, or outcome directly observed in a cited study; inferred implication, meaning a clinically or educationally plausible response not directly outcome-tested in the relevant context; and demonstrated action benefit, meaning a specific action shown to improve a nursing, procedural, or educational outcome. Case reports were used as sentinel evidence for warning signs and high-consequence recognition problems, not as prevalence estimates or proof that a proposed response prevents harm. Most proposed VASC-N behaviours and responses are inferred implications; demonstrated action benefit is limited and does not validate the integrated VASC-N sequence.

### 2.6. Development of the VASC-N Conceptual Framework

Framework development combined inductive and deductive reasoning. Inductively, the evidence chart was examined for repeated decisions, warning signals, adaptations, and communication needs. Deductively, candidate concepts were compared with competency-based education, procedural-safety, ultrasound, documentation, and assessment-validity principles [4,5,12]. All 56 publications were considered; each could inform one or more candidate concepts, but no publication was required to support every domain. Initial charting generated 12 candidate concepts: indication and therapy fit; previous access history; patient knowledge and preferences; inspection and palpation; target identity and patency; depth, diameter, course, and adjacency; dynamic/ultrasound/Doppler verification; site, device, and trajectory adaptation; attempt limits and stop criteria; placement, function, and route confirmation; documentation and handover; and learning, audit, and quality improvement.

A candidate element was retained when it could be expressed as an observable nursing or interprofessional behaviour, had plausible clinical or educational significance, transferred across at least two vascular territories or evidence types, and added non-redundant decision value. Elements were merged when they represented sequential aspects of one decision, and were not retained as independent domains when they were purely descriptive, restricted to one rare territory, not observable, or duplicated another concept. Frequency in the literature was not the sole criterion: a less frequent element could be retained when its potential consequence was high and its response was transferable. The 12 concepts were consolidated into five domains: Verify; Assess; Scan and distinguish; Choose, adapt, and confirm; and Note, notify, and nurture learning.

The author group reviewed the evidence chart, candidate concepts, domain boundaries, terminology, and the proposed Nursing Actionability Test and reporting checklist. Disagreements were resolved by discussion until consensus was reached; no Delphi process, nominal-group technique, formal voting threshold, or external content-validation panel was used. Accordingly, VASC-N, the Nursing Actionability Test, and the checklist are preliminary author-developed conceptual proposals. The consolidation from 12 candidate concepts into five domains is an author-group conceptual organisation; it does not demonstrate that five domains are optimal, mutually exclusive, or psychometrically distinct. External content validation may support retention, revision, merging, or removal of domains. In the acronym, “competencies” denotes proposed observable behaviours and decision processes, not psychometrically validated competencies or a high-stakes assessment instrument.

## 3. Results

### 3.1. Search Yield and Evidence Map

The two searches yielded 2087 records: 656 from PubMed/MEDLINE and 1431 from the Web of Science Core Collection. Exact DOI and normalised-title deduplication removed 404 records, producing 1683 unique records. During purposive selection, 1627 records were not retained for detailed evidence charting; 56 full-text publications published between 1991 and 2026 were charted and included in the narrative synthesis. Thirty-nine publications in the purposive synthesis sample were available only in the Web of Science export, four only in PubMed, and thirteen in both databases. The actual process is shown in Figure 1. The 56-publication set is an information-rich analytical sample rather than a comprehensive or representative corpus.

Interpretive boundary and evidence-status key. Unless a cited primary study directly measured an outcome or association, the practice statements below are author inferences from anatomical plausibility, professional recommendations, or sentinel case evidence. “Recurrent” denotes thematic recurrence within the purposive synthesis, not measured frequency. Three evidence-status categories are used: direct finding (an anatomical feature, association, or outcome directly observed in a cited study); inferred implication (a clinically or educationally plausible response not directly outcome-tested in the relevant context); and demonstrated action benefit (a specific action shown to improve a nursing, procedural, or educational outcome). Most responses proposed below are inferred implications. Descriptive anatomy and case reports do not establish absolute risk, causality, or response effectiveness, and no category or table entry validates the integrated VASC-N sequence.

### 3.2. Exploratory Publication-Context Observation

Within the purposively selected 56-publication synthesis sample, four papers appeared in journals explicitly oriented to nursing or nursing care [31,32,33,34]. This is an exploratory observation about the detailed synthesis sample, not a bibliometric census of the 1683 unique records or of the entire literature. The four papers respectively connected cubital venous formation with puncture-site awareness, an upper-limb arterial variant with injury prevention, persistent left superior vena cava (PLSVC) with interpretation of an apparently malpositioned dialysis catheter, and axillary neurovascular variation with ultrasound-based procedural education.

These examples illustrate—but do not by themselves prove—the translational value of patient-specific vascular anatomy for nursing and health professions education. Anatomical evidence becomes educationally relevant when it may change procedural assessment, site or side selection, vessel or device choice, patient positioning, needle or catheter trajectory, confirmation strategy, monitoring, stop or escalation criteria, documentation and handover, patient counselling, competency design, or a patient- or learner-centred outcome. The exploratory observation should not be read as evidence that only four nursing-relevant publications exist or that journal title perfectly represents readership or professional purpose.

### 3.3. Author-Developed Taxonomy of Patient-Specific Vascular Anatomy

The authors organised the synthesis into four non-equivalent categories: discrete congenital variants, continuous anatomical variability, dynamic anatomical change, and acquired/remodelled anatomy. This is an interpretive taxonomy developed for the present review; it is not a pre-existing or validated classification. Treating all four categories as classical named variants would obscure their different origins and evidentiary meanings. The more useful procedural question is whether the current target is identifiable, patent, accessible, compatible with the intended device or therapy, and safely separated from structures that should not be punctured. Clinical uncertainty signals—such as unexpected pulsatility, non-compressibility, radiating pain, an absent expected vessel, or an unusual line course—are observations that trigger verification; they are not anatomical categories. Table 1 summarises the proposed taxonomy.

### 3.4. Cubital Fossa, Dorsal Hand, and Superficial Venipuncture

The superficial venous system provides the most immediate interface between anatomy and routine nursing practice. In 128 cadaveric cubital fossae, cutaneous nerves crossed above or below the median cubital vein in substantial proportions, and the authors concluded that no single site was safe for every individual; puncture near the cephalic side of the median cubital vein was least likely to injure a nerve [13]. A living study of 100 Brazilian participants (200 limbs) found multiple cubital patterns, with two patterns each accounting for 22%, reinforcing that a familiar “M-shaped” diagram does not describe every patient [14].

Case-based evidence provides a different, lower-level signal. A median cubital vein may travel deep to the bicipital aponeurosis, and venipuncture-related injury of the lateral antebrachial cutaneous nerve can present with immediate electric or radiating pain and persistent symptoms [15,16]. These reports do not estimate event frequency or demonstrate that a particular stop rule reduces injury. They support attention to severe radiating pain as a warning sign, but the response remains an author-inferred, context-dependent implication: clinicians may consider stopping rather than continuing or redirecting blindly and reassessing a vessel whose identity or depth conflicts with inspection and palpation.

Living mapping studies also suggest that initial site selection should be based on the anatomy present rather than a fixed hierarchy of named veins. Infrared assessment of 804 hands identified the fourth intermetacarpal space as the most frequent location of the most prominent dorsal metacarpal vein, whereas a separate study of 722 hands found four cephalic-vein formation patterns but a course through the anatomical snuffbox in 98% [36,37]. Such findings are useful for education and initial assessment, but they should not be converted into a new universal “best vein”. Visibility does not establish compressibility, depth, calibre, absence of nearby artery or nerve, or suitability for the intended therapy.

For nursing practice, the proposed decision process is therefore layered. Inspection and palpation remain proportionate for straightforward venipuncture. Potentially relevant discordant cues include pulsatility, a vessel that does not compress as expected, an unusual trajectory, severe paraesthesia, a rapidly expanding haematoma, or repeated failure despite an apparently suitable vein. A context-dependent response may include increasing anatomical certainty, choosing another site or method, stopping when symptoms suggest neural or arterial contact, and documenting the finding for future care, in accordance with local policy and clinical judgement. The integrated benefit of this response sequence has not been tested.

### 3.5. Upper-Arm Veins, PICCs, Midlines, and Haemodialysis Planning

Upper-arm access exposes the limitations of assuming that the basilic, brachial, and cephalic veins follow a single plan. Preoperative ultrasound mapping in 290 patients (426 arms) found the conventional brachial–basilic arrangement in 66%, while two variant patterns each accounted for 17% [25]. In 494 mapping venograms, bifid cephalic arches occurred in 8.7%, brachial–basilic ladder patterns in 14.0%, and a single brachial vein in 19.3%; 15.7% were considered unsuitable for basilic transposition because early venous confluence could compromise deep drainage [26].

The clinical meaning extends beyond insertion success. A 1004-patient mapping study found cephalic-arch variants in 17.2%; variant anatomy was an independent negative predictor of secondary patency in patients with brachiocephalic fistulas [28]. Reviews and anatomical reports similarly describe upper-limb vascular “traps”, including high-origin arteries, unusual venous confluence, and an ulnar nerve passing through a brachial–basilic venous chiasma, with implications for catheterisation and fistula construction [38,39,40].

For nurses, these findings support consideration—rather than proof of benefit—of two linked practices. First, device choice may be informed by target depth and diameter, availability of a straight intravascular segment, adjacent artery and nerve, intended dwell, infusate, and the need to preserve future access. Second, haemodialysis access may be viewed as longitudinal anatomy rather than a single puncture site. Ultrasound mapping and surveillance can communicate variants, assess maturation, and identify stenosis or thrombosis [27]. Where local pathways support it, a reusable access record may capture cannulation plans, suitable segments, previous infiltrations, aneurysmal areas, and changed thrill or bruit. Whether this combined approach improves clinical or patient-reported outcomes remains to be tested.

This domain also illustrates why a nursing paper should not reduce anatomy to a prevalence table. The more valuable study would test whether a structured map or access plan changes cannulation accuracy, infiltration, pain, access survival, escalation, or patient confidence. Anatomical description becomes operational evidence when it is connected to device–vessel compatibility and longitudinal vessel preservation.

### 3.6. Superficial Arteries and the Risk of Vessel Misidentification

Superficial upper-limb arteries provide a plausible mechanism of avoidable harm when an artery can be mistaken for a vein. Doppler assessment of 314 upper limbs identified a superficial ulnar artery in 2.5% of participants, while a cadaveric series found four superficial ulnar arteries among 95 limbs; both reports emphasised the risk of inadvertent cannulation [17,41]. A clinical ultrasound case specifically highlighted the relevance of a superficially positioned ulnar artery to nursing professionals [42].

Variation is not confined to the ulnar artery. Doppler assessment of 500 upper limbs reported an accessory brachial artery in 15.6%, and a Korean cadaveric study identified superficial brachial arteries in 12.2% of arms [18,19]. A recent meta-analysis estimated a pooled radial-artery variant prevalence of 12%, although heterogeneity was very high and geographic coverage was uneven [20]. Four clinically significant radial-artery anomalies included digital ischaemia after catheter placement in a superficial radial artery and use of the same variant for haemodialysis fistula inflow in other patients [43].

Sentinel reports illustrate possible procedural presentations: a “missing” radial pulse can reflect an altered arterial course, and a persistent median artery can be mistaken for the intended radial artery during catheterisation; in the latter case, ultrasound exposed both the variant vessel and its relationship to the median nerve [44,45]. A nursing-journal case similarly argued that appreciation of upper-extremity arterial variants is essential to prevent injury [32].

The practical principle proposed from this synthesis is identity before entry. Palpation is useful but not infallible, and colour alone is not diagnostic. Potential warning signs include unexpected pulsatility, bright or forceful backflow, pain, resistance, distal colour or temperature change, altered capillary refill, or an unusual vessel course. These cues may justify immediate reassessment. When arterial identity, collateral supply, or distal perfusion is uncertain, stopping and escalating in accordance with local policy may be considered instead of repeated cannulation. These responses are clinically plausible and partly aligned with professional safety principles, but their effectiveness as an integrated VASC-N pathway has not been tested. Future nursing research could evaluate recognition cues, confirmation methods, response time, neurovascular assessment, and patient outcomes rather than arterial prevalence alone.

### 3.7. Internal Jugular Vein and Central Venous Access

The internal jugular vein (IJV) provides the clearest evidence that landmark anatomy is probabilistic rather than deterministic. Early ultrasonographic work documented clinically important deviations in IJV position that could explain difficult central access [21]. Subsequent studies showed that the IJV may be lateral, anterolateral, or anterior to the common carotid artery, and that head rotation can increase vessel overlap rather than improve safety [22,23,46].

Patient factors and scanning level also matter. In a visual ultrasound study at three neck levels, body mass index correlated with the depth of both the IJV and the carotid artery, and anatomical variability was greater than anticipated [47]. A systematic review and meta-analysis estimated an overall IJV-variant prevalence of 3.36%, but heterogeneity exceeded 94%, demonstrating that a single prevalence estimate cannot substitute for preprocedural assessment [48].

The evidence in uraemic patients is particularly relevant to nursing and dialysis pathways. An ultrasonographic survey reported occult IJV variations capable of complicating temporary haemodialysis access, and a later case demonstrated that Doppler assessment can identify a variant IJV position before puncture rather than after a failed or harmful attempt [49,50]. These findings support consideration of preprocedural scanning when central access is planned, anatomy is uncertain, previous attempts have failed, thrombosis is possible, or the consequence of error is high. They do not demonstrate that a VASC-N-based scanning decision improves outcomes.

Ultrasound guidance is not synonymous with simply placing a probe on the neck. Reviews and a professional position statement distinguish preprocedural assessment from real-time needle guidance and emphasise vessel size, depth, thrombosis, anatomical variation, needle-tip visualisation, and guidewire confirmation [24,51,52]. Static skin marking cannot control a needle after the patient, vessel, or probe moves. These sources describe relevant competency elements—cognitive anatomy, equipment operation, asepsis, image optimisation, dynamic tip control, complication recognition, and an escalation pathway—but they do not validate the VASC-N domain or establish that every setting requires the same approach.

### 3.8. Axillary and Femoral Access: Overlap, Depth, and Dynamic Anatomy

Axillary and subclavian access demonstrate the interaction between fixed relationships and dynamic physiology. In 150 cardiac-surgery patients, the right axillary vein was larger in 69%; the vein lay directly over the artery in 67% at a midclavicular view, but only 7% at a more lateral view; and 4% showed an aberrant position [30]. A prospective study of 110 patients found that axillary-vein area increased after transition to controlled mechanical ventilation, illustrating that vessel calibre and collapsibility change with intrathoracic pressure [53].

Femoral anatomy is similarly variable. Bedside ultrasonography in 180 adults showed that the amount of femoral vein not overlapped by the artery decreased at more distal landmark levels, and some patients lacked an exposed venous segment at all assessed levels [29]. In 254 patients undergoing ultrasound-guided femoral puncture, high-overlap and lateral-vein patterns were substantially more frequent at the lower inguinal level; the reported vascular-complication rate was 0.4% [54]. A variant deep femoral artery may even pass anterior to and spiral around the femoral vein, creating a mechanism for arterial injury or arteriovenous fistula during venous cannulation [55].

Venous duplication adds another layer. A systematic review estimated pooled femoral-vein duplication at 19.7%, with much higher prevalence in imaging than cadaveric studies, while a large anatomical series found non-modal femoral venous patterns in 12% of limbs [56,57]. In patients with renal failure, ultrasound also identified depth and suitability problems that were not reliably resolved by surface palpation, particularly in obesity [58].

For nursing pathways, the implication is not that every femoral or axillary access must be performed by the same professional group. Rather, nurses who prepare, assist, monitor, use, or troubleshoot these devices may need to understand why site, patient position, ventilation, body habitus, and dynamic ultrasound can matter. Post-procedural swelling, pain, bleeding, limb perfusion change, or device dysfunction may warrant interpretation in relation to variable anatomy and escalation according to local protocols. This is a clinically plausible application rather than evidence that a VASC-N pathway improves outcomes.

### 3.9. Unexpected Central Catheter Course and Persistent Left Superior Vena Cava

An anomalous thoracic venous route creates a different safety problem: a catheter may be intravascular yet appear “malpositioned” on a chest radiograph. PLSVC has been estimated in approximately 0.3–0.5% of otherwise healthy individuals and is often discovered only after a left-sided central catheter follows an unexpected course [59]. A review of misplaced central catheters emphasised that congenital variants, thrombosis, stenosis, and true extravascular placement require different management; removal should not proceed until the route and vulnerable structures are understood [60].

Dialysis and vascular-access cases show both sides of the problem. Some PLSVC catheters have functioned after imaging confirmed an acceptable intravascular route, and authors warned against alarming misinterpretation or unnecessary manipulation [33,61,62]. Conversely, other cases involved coronary-sinus positioning, thrombosis, mediastinal haematoma, or associated absence of the right IJV, demonstrating why post-procedural imaging and multidisciplinary interpretation remain essential [63,64].

PLSVC can also be discovered during PICC placement through an unexpected electrocardiographic or radiographic pattern; one report recommended a specialised patient profile so that the anatomy would be available for future care [65]. More generally, even without PLSVC, the angle of the left IJV–brachiocephalic axis has been associated with haemodialysis catheter dysfunction, reinforcing that central route geometry can affect device performance [66].

This domain is especially nursing-relevant because a consequential decision may occur after insertion: whether to use, manipulate, withdraw, exchange, or immobilise the device; what monitoring is required; and whom to notify. An unusual line course is not a diagnosis. An author-proposed, context-dependent response is to consider withholding use when function or route is uncertain, securing the catheter, assessing the patient, obtaining definitive imaging or expert interpretation, and preserving confirmed anatomical information in the record, subject to local policy and authorised clinical review. This sequence is supported primarily by applied reviews, professional practice principles, and sentinel cases; its outcome benefit as part of VASC-N has not been tested.

### 3.10. Injection-Related Evidence: A Delimited Gap

The database strategies deliberately included injection terms because nurses commonly administer intramuscular and subcutaneous medications. However, the retrieved records that combined vascular variation with injection were dominated by cosmetic, regional-anaesthesia, operative, or specialty contexts and did not provide a coherent evidence base for routine nurse-administered injections. The axillary study in a nursing journal showed that neurovascular relationships may depart from textbook expectations, but its direct context was peripheral nerve block rather than routine injection practice [34].

This gap should not be concealed by importing unrelated anatomical claims. It indicates a separate research agenda: living ultrasound studies of vessel, nerve, muscle, and subcutaneous-tissue relationships at commonly used injection sites; stratification by age, sex, body composition, frailty, prior surgery, and positioning; and evaluation of whether anatomy-informed site or needle-length decisions reduce pain, failed delivery, bleeding, or neural injury. Until such evidence is available, the present framework applies to injection only at the level of assessment, uncertainty recognition, stop rules, and escalation—not as a source of unvalidated site prescriptions.

### 3.11. Cross-Cutting Mechanisms Linking Patient-Specific Vascular Anatomy to Potential Harm

Across the anatomical regions and procedural contexts represented in the purposive synthesis, the authors organised the evidence around six recurring interpretive mechanisms through which patient-specific vascular anatomy may contribute to procedural risk in nursing practice. First, potential target misidentification occurs when an artery, thrombosed vein, nerve-adjacent structure, or anomalous vessel is mistaken for the intended target. Second, unsafe adjacency may place a nerve or artery within the needle path even when the intended vein is correctly identified. Third, device–vessel mismatch may arise when vessel depth, diameter, curvature, or route is incompatible with the selected catheter, therapy, or dwell. Fourth, dynamic change may make a static assessment obsolete. Fifth, an anomalous central route may be misinterpreted as technical malposition or used before confirmation. Sixth, useful patient-specific anatomical knowledge may be lost between clinicians or encounters, recreating avoidable uncertainty. These are qualitative synthesis categories, not measured causal pathways or frequency estimates.

Table 2 presents these mechanisms as an evidence-to-action map with explicit evidence-status labels. The table separates direct anatomical or empirical findings from clinically plausible author-inferred implications and from any demonstrated action benefit. The proposed responses are context-dependent options unless a demonstrated benefit is expressly identified. An anatomical pattern does not dictate one universal action; it may alter the probability, consequence, or information required for the next decision.

### 3.12. The VASC-N Conceptual Framework

**Figure 2:**
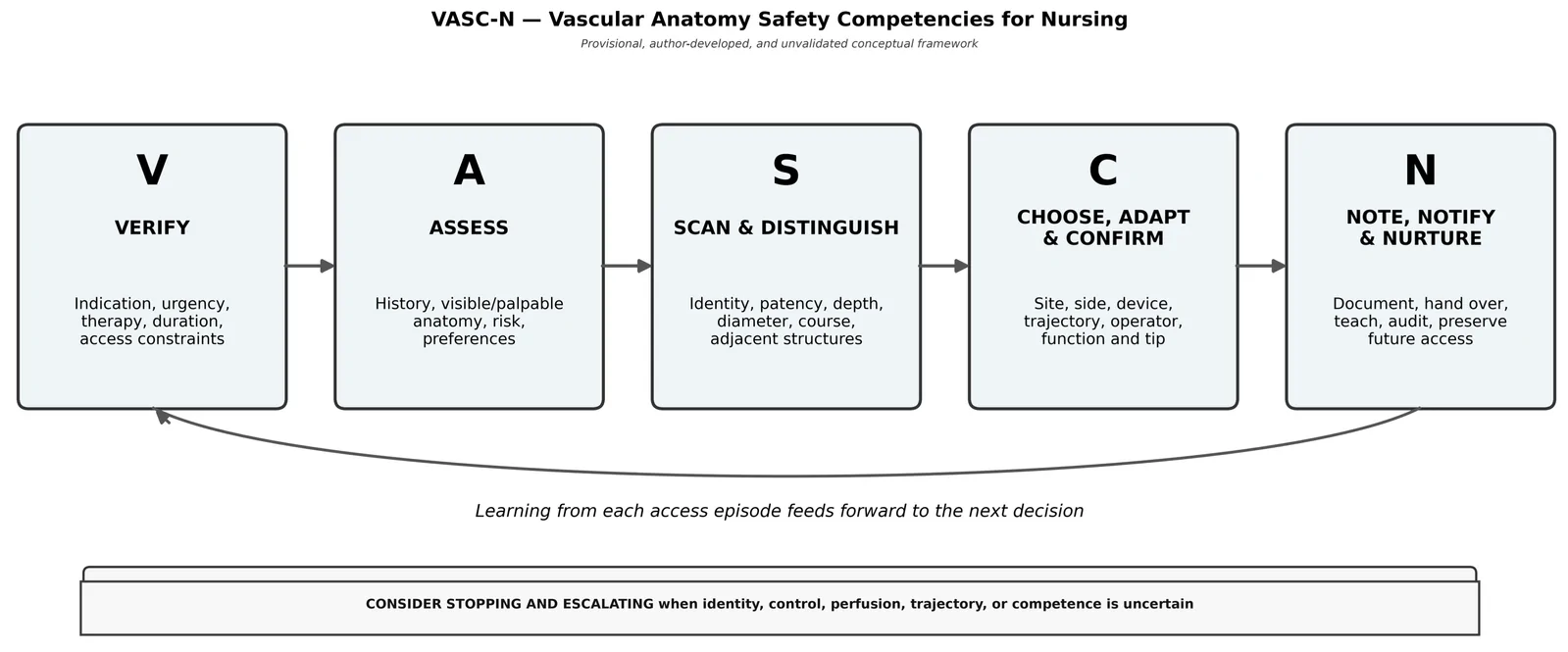
VASC-N (Vascular Anatomy Safety Competencies for Nursing). The five provisional domains form an author-developed learning cycle: information documented after one access episode may inform the next decision. “Competencies” denotes proposed observable behaviours. The domain structure may change after external validation, and VASC-N has not been validated as a competency instrument or shown to improve safety or learning outcomes.

VASC-N stands for Vascular Anatomy Safety Competencies for Nursing. Pending validation, “competencies” refers to proposed observable behaviours and decision processes rather than validated psychometric competencies. VASC-N is an author-developed five-domain conceptual cycle, not a catalogue of variants or a directive that one profession independently perform every procedure represented in the evidence. The consolidation into five domains does not establish that their number or boundaries are optimal or psychometrically distinct; external content validation may justify revision, merging, or removal. The cycle begins before a device is selected and continues after placement and handover. It is proposed for proportionate formative application to routine venipuncture and for potential extension to difficult, deep, central, arterial, or haemodialysis access. The provisional five-domain cycle is depicted in Figure 2, and the proposed questions, behaviours, stop or escalation triggers, and evidence status are presented in Table 3.

The “Scan and distinguish” domain represents a proposed graded sequence. Visual assessment and palpation may remain proportionate for many straightforward peripheral procedures. Dynamic assessment adds repositioning, compression, and observation of change. Preprocedural B-mode ultrasound may be used when indicated to assess identity, patency, depth, diameter, course, and adjacency; Doppler adds flow information; real-time ultrasound guidance addresses needle or device control during entry; and post-procedure imaging addresses tip or route questions. These activities are distinct, require different training, and should not be interpreted as a recommendation for universal ultrasound use. Their integration within VASC-N remains unvalidated.

Any application would also need to be role-specific. Depending on jurisdiction and local policy, a nurse may: (1) perform routine procedures independently; (2) perform advanced access procedures only after additional education, credentialing, and supervised competency assessment; (3) assist another authorised operator; or (4) primarily recognise a problem, monitor the patient or device, stop or escalate care, and document and communicate patient-specific anatomy. These are proposed role categories rather than validated competency levels. VASC-N addresses decision quality across these roles and does not expand professional scope.

### 3.13. Preliminary Nursing Actionability Test for Publication

An anatomical finding is not automatically suited to a nursing or health professions education journal merely because it is unusual. The author-proposed Nursing Actionability Test asks whether a manuscript completes an anatomy-to-action chain: it defines the anatomical construct, establishes how it was observed, names the nursing or interprofessional task, explains whether a failure/harm mechanism is measured or only plausible, identifies a recognition cue, specifies a feasible adaptation or escalation, and measures an outcome relevant to patients, devices, clinicians, learners, education, or services. Failure at one link does not invalidate a study, but it weakens an education-focused rationale. The test is a preliminary conceptual prompt, not a validated appraisal instrument or editorial acceptance rule.

The preliminary test also distinguishes awareness from observable performance. “Learners should be aware” is not an adequate endpoint unless a manuscript explains what awareness may change. An educational implication may be expressed as a testable decision or behaviour, such as selecting another site, changing the device or length, repositioning the patient, verifying vessel identity, stopping for paraesthesia, obtaining imaging, withholding catheter use pending confirmation, escalating to a vascular-access specialist, documenting an anatomical alert, or teaching a specific recognition-and-response sequence. These examples are proposed research and assessment targets, not evidence that the actions improve outcomes. The principal publication purposes, suitable study designs, and priority outcomes are summarised in Table 4.

## 4. Discussion

### 4.1. Principal Interpretation

This review identified a clinically diverse anatomical literature but only four publications in the purposive synthesis sample that appeared in explicitly nursing-oriented journals [31,32,33,34]. This sample-specific observation is exploratory and cannot be extrapolated to the entire literature. The more defensible finding is a recurrent translational discontinuity across evidence types: anatomy studies typically establish morphology or prevalence, procedural studies examine access or device outcomes, and educational studies often assess generic technical performance. The missing layer is frequently the explicit chain from patient-specific anatomy to recognition, decision adaptation, communication, and a measurable patient-, clinician-, learner-, or service-level outcome.

A central conceptual conclusion is that patient-specific vascular anatomy is not limited to rare named congenital anomalies. Continuous, dynamic, and acquired or remodelled anatomy may be more common and immediately relevant to decisions: vessel depth and diameter affect device fit, carotid–IJV overlap changes with level and head position, axillary calibre changes with intrathoracic pressure, and haemodialysis circuits remodel with maturation, stenosis, thrombosis, and prior cannulation [21,22,23,24,25,26,27,28,29,30,47,53,58]. Discrete arterial and venous variants remain important when they alter identity, pulse location, route, or collateral supply [17,18,19,20,41,42,43,44,45,48,55,56,57,58,59,60,61,62,63,64,65,66]. These are direct anatomical findings or associations; they do not, by themselves, prove that a particular educational or clinical response reduces complications. The implication that they should alter a decision is generally inferred and must be tested. VASC-N therefore treats variability as a condition for decision-making rather than as a list to memorise.

Ultrasound should be understood as a family of distinct anatomical and procedural activities rather than as one undifferentiated intervention. Visual assessment, palpation, dynamic repositioning or compression, preprocedural B-mode identification, Doppler assessment, real-time needle guidance, and post-procedural route or tip imaging answer different questions [24,47,49,50,51,52,59,60,61,62,63,64,65]. Procedure-specific evidence supports ultrasound in defined vascular-access settings, particularly central access and difficult peripheral access, but this evidence does not validate VASC-N or support mandatory ultrasound for every routine venipuncture. The proposal that education address indications, interpretation, dynamic tracking, stop criteria, documentation, and escalation as well as image acquisition is therefore an inferred educational implication requiring evaluation.

Documentation is a plausible component of anatomical safety and continuity, although its independent effect remains insufficiently tested. Patients with difficult access may know which site, device, position, or operator has worked previously, and case reports of anomalous central routes recommend persistent patient-specific records or alerts [33,59,60,61,62,63,64,65,66]. Failure to capture this information may recreate uncertainty at later encounters, but evidence that structured access notes reduce failed attempts or improve experience is lacking. VASC-N therefore includes “Note, notify, and nurture learning” as an inferred component of the proposed cycle; prospective studies must determine whether it improves continuity, experience, learning, or outcomes.

The strength and directness of the evidence are uneven. Within the three evidence-status categories, living imaging and cadaveric studies principally provide direct anatomical findings but generally do not test nursing outcomes. Cohort studies can provide measured associations, yet residual confounding limits causal interpretation. Professional recommendations support defined practices but were not developed to validate VASC-N. Case reports and small series are valuable as sentinel learning units because they reveal warning signs and possible mechanisms; they cannot determine event frequency, absolute risk, or response efficacy. Most VASC-N actions therefore remain inferred implications. Demonstrated action benefit is limited to specific procedures or educational interventions and cannot be transferred automatically to the integrated framework. Anatomical plausibility, clinical relevance, and educational-intervention effectiveness must remain analytically distinct.

### 4.2. Implications for Daily Nursing and Interprofessional Practice

Variation-aware practice does not require every nurse or learner to diagnose every named arterial or venous variant. The framework proposes a role-appropriate response to uncertainty. For straightforward venipuncture, a context-dependent approach may include history, inspection, palpation, a clear target, a controlled needle path, and consideration of stopping for severe radiating pain or unexpected arterial features [13,14,15,16,17,18,19,20]. For predicted difficult access, deep upper-arm vessels, central or arterial procedures, haemodialysis circuits, or an unusual catheter route, consideration of imaging, an experienced operator, specialty review, or escalation should be guided by procedure-specific evidence, local policy, available resources, and professional scope [21,22,23,24,25,26,27,28,29,30,41,42,43,44,45,46,47,48,49,50,51,52,53,54,55,56,57,58,59,60,61,62,63,64,65,66]. The proposed educational aim is calibrated judgement: learners may need to distinguish what they can proceed with, what requires additional credentialing or supervision, what they can assist with, and what they should recognise, monitor, escalate, or document. These integrated behaviours require validation.

Attempt limits are a plausible anatomical as well as service-safety consideration. Repeating the same blind method after failure assumes that the target exists where expected and that additional persistence will solve the problem. Venous depth, small calibre, overlap, thrombosis, anomalous course, or unsafe adjacency may instead make the original plan structurally unsuitable [15,16,21,25,29,47,49,50,58]. VASC-N interprets failure as information: the anatomy, device, patient position, operator skill, or route may be mismatched. An author-proposed next step is to increase certainty or change the plan rather than merely repeat it. This principle should be tested in simulation and practice through explicit attempt-limit and escalation outcomes, not inferred from eventual cannulation success alone [5,6,7,8].

Vessel preservation is another plausible decision consideration. A technically successful catheter may still be suboptimal if it consumes a critical future site, occupies too much of a small vein, traverses an unsuitable route, or delays a more appropriate device. Upper-arm mapping, cephalic-arch variants, and haemodialysis-access literature show associations among anatomy, suitability, patency, dysfunction, and long-term access options [25,26,27,28,38,39,58,66]. VASC-N therefore proposes linking the immediate procedure to the whole therapy plan and, in patients with kidney disease, to future dialysis access. This longitudinal linkage is conceptually suitable for interprofessional teaching, but its effect on access preservation or outcomes has not been demonstrated.

The patient may also provide relevant anatomical and procedural information. Prior painful attempts, known variants, successful ultrasound-guided access, protected limbs, fistulas, thrombosis, or a history of lines following an unusual route can inform the plan [33,59,60,61,62,63,64,65,66]. Asking, explaining, and documenting these experiences is consistent with person-centred practice, but the independent outcome benefit of incorporating them into a VASC-N pathway remains untested. Educational cases could include patient-provided information so that learners can be assessed on how they consider it in site selection, consent, escalation, and a reusable access plan.

### 4.3. Implications for Nursing and Health Professions Education

The educational implications extend beyond procedural nursing. Anatomy is retained and valued when learners can connect structure to clinical purpose [1,2], and variant anatomy literacy has been framed as a patient-safety competency in surgical training [3]. Focused ultrasound teaching has enabled novice medical learners to identify major neck vessels and their variability, while systematic reviews support ultrasound as a means of connecting living anatomy with examination and procedural reasoning [9,10,11,67]. Simulation studies further show that deliberate practice and mastery learning can improve procedural performance and, in central venous catheterisation, reduce patient complications [6,7,8,68]. Automated analysis of clinical ultrasound recordings may also help build simulation libraries that reflect genuine anatomical diversity rather than a single idealised manikin [69].

Nursing and health professions curricula may usefully teach three linked layers. The first is canonical anatomy: names, usual relationships, and landmarks. The second is patient-specific variability: the expectation that number, depth, course, adjacency, patency, and dynamic behaviour differ among patients and encounters. The third is uncertainty management: how to verify, adapt, stop, escalate, communicate, and preserve information within one’s role and local scope. Teaching canonical anatomy without variability risks false certainty; teaching variability without an action pathway produces interesting but non-operational knowledge. This layered proposal aligns conceptually with competency-based education and Miller’s progression [4,5], but it has not been evaluated as a curriculum.

Assessment could extend beyond successful cannulation on a uniform manikin. Future curricula might include cases with a superficial artery, a vein overlying a nerve, a deep small target, artery–vein overlap, thrombosis, a vessel that collapses under probe pressure, an unexpected central catheter course, and a patient who reports a prior access plan [13,14,15,16,17,18,19,20,21,22,23,24,25,26,27,28,29,30,41,42,43,44,45,46,47,48,49,50,51,52,53,54,55,56,57,58,59,60,61,62,63,64,65,66]. Candidate performance criteria may include target identification, device choice, verbalised risk, dynamic needle-tip control, stop behaviour, escalation, confirmation, documentation, and communication with the patient. Simulation evidence supports repeated practice with feedback and mastery standards [6,7,8], but a VASC-N-specific assessment claim should not be made until evidence supports the content, response process, internal structure, relationships with other variables, and consequences of the intended use [12].

VASC-N may be tested initially as a formative debrief structure and curriculum map. Educators could ask what the learner verified, which patient and anatomical factors were assessed, how the target was distinguished, what was adapted and confirmed, and what information might be carried forward. Table 3 presents the provisional domains, while Table A2 provides proposed bedside, simulation, and competency prompts, and Table A1 provides a preliminary reporting checklist. This approach is intended to link bioscience with clinical judgement and make escalation observable; it has not been shown to improve learning and should not be used for high-stakes assessment until appropriate validity evidence is established [12].

### 4.4. Implications for Education Research and Publication

Future studies could move beyond prevalence-only reporting and explicitly test the anatomy-to-action chain. High-value questions include whether a mapped feature changes the selected site or device; whether a structured pre-scan reduces attempts or complications in an indicated population; whether anatomical documentation prevents recurrent difficult access; whether simulation with variant cases improves recognition and escalation; and whether patient-reported access histories improve planning. Prospective observational studies, diagnostic-accuracy studies, pragmatic trials, mixed-methods research, implementation evaluations, and multicentre registries can address different links. Educational studies should predefine whether the intended outcome is knowledge, simulated performance, workplace behaviour, patient outcome, or system effect, and should not treat satisfaction or confidence as evidence of competence [4,5,6,7,8,12].

Rigorous reports would state whether anatomy was established by dissection, ultrasound, Doppler, computed tomography, magnetic resonance imaging, surgical observation, fluoroscopy, or post-procedural imaging; describe patient position and physiological conditions; provide denominators and laterality; distinguish congenital from acquired anatomy; and report observer training or reliability when classification is subjective. A phrase such as “abnormal anatomy” is insufficient. The need for transparent search, reasoning, and endpoint reporting is consistent with SANRA [35], while the clinical literature demonstrates that prevalence and apparent relationships vary materially with ascertainment method, scanning level, position, and population [21,22,23,24,25,26,27,28,29,30,47,48,53,54,55,56,57,58].

The nursing and educational context would ideally identify who performed or learned the procedure, their prior training and scope, the setting and urgency, the intended therapy and device, available escalation resources, the comparator, and the outcome that could plausibly change. The four nursing-focused publications in the purposive synthesis sample illustrate this translational requirement by connecting anatomical findings to puncture-site awareness, arterial-injury prevention, catheter-route interpretation, or ultrasound-based education [31,32,33,34]. Authors should avoid implying that awareness alone prevents complications; the intervention or decision pathway connecting recognition to outcome must be visible and testable.

Case reports remain valuable when an event is rare but consequential, especially when they document the cue, immediate response, confirmatory imaging, multidisciplinary reasoning, patient outcome, and prevention of recurrence [15,16,32,33,42,43,44,45,50,55,59,61,62,63,64,65]. However, a case should not overstate prevalence, causality, or generalisability. In a nursing or health professions education journal, it may function as a sentinel learning unit: authors could specify what a learner or clinician might recognise, which response was actually taken or is merely proposed, how performance could be assessed, and how the finding could be incorporated into simulation, policy, or documentation.

### 4.5. Framework Implementation and Validation Agenda

VASC-N must be treated as a testable conceptual model rather than an established competency or safety intervention. A first validation stage could establish content validity with nurses, physicians, vascular-access specialists, anatomists, educators, patients with difficult access, nephrology teams, radiologists, and other clinicians who insert, assist with, monitor, or manage vascular devices. Cognitive interviews could test whether the domains and prompts are understandable, whether “Scan” is interpreted proportionately rather than as universal ultrasound, whether role and scope distinctions are clear, and whether important behaviours are missing. Evidence should be gathered for each proposed interpretation and use rather than equating expert agreement with validation [12].

A second stage could test feasibility, response processes, and reliability. Candidate indicators include completion of risk history, use of an appropriate attempt limit, identification of a clear target, documentation of ultrasound findings when used, time to escalation, confirmation of function or route, and completeness of the reusable access note. Think-aloud studies and direct observation can determine how users interpret each item; inter-rater agreement, internal structure where applicable, and administrative burden should be measured before VASC-N is converted into a checklist or competency instrument [12].

A third stage could evaluate educational transfer and clinical outcomes in stepped-wedge, cluster, or pragmatic implementation studies. Relevant endpoints include recognition accuracy, decision quality, first-pass and overall success, total punctures per episode, pain and anxiety, arterial or neural injury, infiltration, time to therapy, device dwell, thrombosis, access preservation, unnecessary catheter manipulation, patient trust, and equity across age, body habitus, comorbidity, skin tone, and prior access history [6,7,8,13,14,15,16,17,18,19,20,21,22,23,24,25,26,27,28,29,30,41,42,43,44,45,46,47,48,49,50,51,52,53,54,55,56,57,58,59,60,61,62,63,64,65,66]. The framework should be revised, merged, simplified, or abandoned if external validation does not support the five-domain structure or if implementation adds burden without improving decisions, behaviour, or outcomes.

### 4.6. Strengths and Limitations

This review used two complementary databases with native export files, reported exact yields and provenance, applied reproducible exact DOI or title deduplication, and reproduced the search strategies. The author-developed taxonomy, evidence-to-action map, VASC-N framework, preliminary Nursing Actionability Test, and reporting checklist were constructed from decision points spanning superficial, peripheral, central, arterial, femoral, axillary, thoracic, haemodialysis, and educational contexts. These outputs are conceptual organisations of a purposive sample, not validated classifications or comprehensive evidence maps. The manuscript was structured with reference to SANRA [35] and anchored in established work on anatomy education, competency-based education, simulation, ultrasound teaching, and assessment validity [1,2,3,4,5,6,7,8,9,10,11,12].

The limitations materially constrain interpretation. This was a targeted narrative review with single-reviewer initial inspection and purposive selection, not a systematic or scoping review with duplicate independent screening. A separate prospectively logged full-text exclusion stage and reason-by-reason exclusion count were not maintained. The 56 publications should not be interpreted as a comprehensive, exhaustive, or representative sample of all potentially eligible literature; their composition reflects the selected databases, search terminology, single-reviewer judgement, purposive information-rich criteria, author-group interpretation, and publication patterns. Although explicit criteria, standardised charting, and author-group review increased transparency, selection bias, interpretive subjectivity, and limited reproducibility remain. Publication bias may favour unusual or consequential variants and successful educational or procedural reports. Terminology bias may have excluded records that did not use nursing, procedural, or conventional anatomical-variation terms.

Database coverage was also limited. PubMed/MEDLINE and the Web of Science Core Collection were searched, but CINAHL, ERIC, Embase, and Scopus were not; the strategy may therefore underrepresent nursing, allied-health, education, non-English, or differently indexed evidence. No universal risk-of-bias instrument was applicable across the heterogeneous designs, and many studies were cadaveric, single-centre, observational, or case-based. Anatomical differences and plausible mechanisms should not be equated with proven complication reduction, and sentinel cases cannot quantify absolute risk or treatment effect. The observation that four papers appeared in nursing-oriented journals refers only to the purposive synthesis sample. Finally, VASC-N, its five-domain organisation, the Nursing Actionability Test, the taxonomy, and the reporting checklist have not undergone external content validation, feasibility testing, reliability assessment, educational-transfer evaluation, or clinical-outcome testing. Domain boundaries may require revision, merging, or removal.

## 5. Conclusions

Patient-specific vascular anatomy may become relevant to nursing and health professions education whenever a clinician or learner must identify a vessel, choose a device, cross tissue with a needle, interpret blood return or imaging, monitor a catheter, respond to pain or perfusion change, preserve future access, or communicate what occurred. The most informative future publications will move beyond showing that anatomy differs: they will specify which decision may change, why, how the associated behaviour can be taught and assessed, and whether the change improves a patient-, clinician-, learner-, device-, or service-level outcome.

VASC-N (Verify; Assess; Scan and distinguish; Choose, adapt, and confirm; and Note, notify, and nurture learning) offers an author-proposed practical sequence for translating anatomical uncertainty into action-oriented assessment and performance. Neither the individual domains nor the integrated sequence provide evidence of safer care, greater procedural success, or more effective learning. When observed anatomy does not support the expected procedure, the proposed, context-dependent options may include increasing anatomical certainty, adapting the plan, stopping or escalating when control or competence is inadequate, confirming function or route, and preserving relevant information for the next encounter. These options require testing within defined professional roles and clinical contexts.

Patient-specific vascular-anatomy manuscripts for nursing and health professions education may be strengthened by integrating rigorous anatomical ascertainment with an explicit professional or educational problem, observable action, assessment strategy, person-centred outcome, documentation plan, evidence-status statement, and transparent account of limitations. Prospective validation is required before claiming that VASC-N or the accompanying tools improve recognition, procedural reasoning, escalation, learning transfer, access preservation, patient experience, procedural success, or clinical safety.

## Figures and Tables

**Table 1:** Author-developed taxonomy of patient-specific vascular anatomy for nursing practice and health professions education.

Category	Definition	Examples	Potential Nursing Relevance	Minimum Reporting Implication
Discrete congenital variant	Altered origin, number, branching, persistence, absence, or course.	Superficial ulnar artery; accessory brachial artery; duplicated vein; PLSVC.	May alter target identity, pulse location, catheter route, or collateral supply.	Name the exact pattern, laterality, denominator, and confirmatory method.
Continuous anatomical variability	A measurable distribution rather than a categorical anomaly.	Depth, diameter, tortuosity, mobility, vessel-to-nerve distance, vessel overlap.	May affect needle/catheter fit, stability, adjacency, or dwell suitability.	Report distributions and clinically meaningful thresholds; avoid over-labelling extremes.
Dynamic anatomical change	Anatomy changes with position or physiological/procedural conditions.	Head rotation, limb position, ventilation, compression, venous filling, probe pressure.	A target may narrow, move, collapse, or overlap another structure during assessment or access.	Specify position and physiological conditions; use dynamic assessment when relevant.
Acquired or remodelled anatomy	Vascular configuration altered by disease or previous intervention.	Thrombosis, stenosis, scarring, catheters, surgery, fistula/graft, collaterals.	Landmarks may become unreliable and future access options may require preservation.	Separate acquired from congenital anatomy and report relevant procedural history.

This taxonomy is an author-developed interpretive organisation and has not been validated. Categories may coexist in one patient or encounter; uncertainty signals are handled as triggers for verification rather than as a fifth anatomical category.

**Table 2:** Evidence-to-action map with explicit separation of direct findings, inferred implications, and demonstrated action benefits.

Clinical Domain	Representative Evidence	Evidence Status and Source	Potential Failure or Harm	Author-Proposed, Context-Dependent Response	Candidate Outcomes
Cubital fossa and hand	Variable venous patterns; nerve–vein crossings; deep median cubital vein; dorsal-hand/cephalic formation [13,14,15,16,36,37].	Direct finding: Cadaveric and living mapping plus sentinel cases. Inferred implication: Verification, stopping, and documentation responses. Demonstrated action benefit: Not established for the integrated response.	Nerve or arterial injury, haematoma, repeated failure, pain.	May include inspection and palpation in procedural position; avoidance of blind redirection; consideration of stopping for radiating pain; verification of ambiguous identity or depth; and documentation of relevant sites.	Attempts; neurological symptoms; haematoma; pain; patient confidence.
Peripheral and upper-arm access	Variant brachial–basilic confluence, paired/single brachial veins, cephalic-arch variants, venous–neural chiasma [25,26,28,40].	Direct finding: Mapping studies, a cohort association, and anatomical case evidence. Inferred implication: Anatomy-device matching, access preservation, and escalation. Demonstrated action benefit: The integrated pathway has not been tested.	No controlled catheter path; device–vessel mismatch; infiltration; thrombosis; access loss.	May include mapping identity, diameter, depth, course, patency, and adjacency; considering device fit and future access; and escalating when no controlled route is identified.	Device selection; occupancy; infiltration; thrombosis; dwell; access survival.
Superficial arteries/arterial access	Superficial ulnar/brachial/radial artery, accessory brachial artery, persistent median artery [17,18,19,41,43,44,45].	Direct finding: Living and cadaveric mapping plus sentinel cases. Inferred implication: Layered identity checks and escalation. Demonstrated action benefit: Not established; absolute risk remains unknown.	Inadvertent arterial entry/injection, haematoma, nerve contact, distal ischaemia.	May include layered identity checks; Doppler or ultrasound for conflicting cues; distal assessment; and stopping or escalating suspected injury according to local policy.	Recognition time; arterial injury; perfusion; neurological outcome; rescue intervention.
Internal jugular/central access	Variable internal jugular vein position, depth, calibre, carotid overlap, thrombosis, and response to head rotation [21,22,23,47,49].	Direct finding: Prospective and cross-sectional ultrasound studies. Inferred implication: Context-specific pre-scan, real-time guidance, and confirmation, supported by professional recommendations. Demonstrated action benefit: Varies by ultrasound context and does not validate VASC-N as a whole.	Arterial puncture, repeated passes, haematoma, guidewire difficulty, failed access.	May include a pre-scan at the intended level and position, real-time guidance when indicated, tip or guidewire visualisation, and avoidance of excessive rotation.	Passes; arterial puncture; time; success; complications; demonstrated competency.
Axillary and femoral access	Variable overlap, depth, lateral/posterior position, duplication, and ventilation-related calibre change [29,30,53,54,55,56].	Direct finding: Observational ultrasound, anatomical studies, and meta-analysis. Inferred implication: Dynamic assessment and post-procedural monitoring. Demonstrated action benefit: The integrated response is untested; most outcomes are surrogate or associated.	Arterial puncture, haematoma, false landmark confidence, device dysfunction.	May include dynamic assessment in the actual position, selection of a level with a controlled window, and monitoring of bleeding, swelling, perfusion, and function.	Complications; exposed-vein window; first-pass success; function; escalation.
Central venous route/PLSVC	Persistent left superior vena cava associated with an absent internal jugular vein, stenosis/thrombosis, and unusual left-sided geometry [59,60,61,62,63,64,65,66].	Direct finding: Applied reviews, case reports or series, and one cohort association. Inferred implication: Withholding use pending confirmation, avoiding blind removal, and preserving an anatomical alert. Demonstrated action benefit: Not established; cases are sentinel, not risk estimates.	Route misclassification, unsafe use/removal, arrhythmia, thrombosis, mediastinal injury, dysfunction.	May include withholding use when route or function is uncertain, securing the device, obtaining definitive imaging or expert review, avoiding blind removal, and preserving a persistent anatomical alert.	Correct interpretation; time to confirmation; adverse events; unnecessary manipulation; future reuse of information.
Haemodialysis access	Created circuit, variant inflow/outflow, accessory veins, stenosis, aneurysm, depth, and arch anatomy [27,28,38,39].	Direct finding: Imaging review, mapping studies, cohort association, and anatomical reports. Inferred implication: Longitudinal mapping, segment avoidance, and escalation. Demonstrated action benefit: The integrated pathway requires prospective testing.	Difficult cannulation, infiltration, prolonged bleeding, thrombosis, access abandonment.	May include use and updating of a longitudinal access map, avoidance of unsuitable segments, and escalation of changed thrill or bruit, recurrent infiltration, or poor performance.	Cannulation accuracy; pain; dialysis adequacy; patency; access longevity.
Education and simulation	Living anatomy differs from textbook/manikin assumptions; ultrasound and automated analysis can diversify cases [34,67,68,69].	Direct finding: Observational education, a small simulation intervention, and imaging-analysis studies. Inferred implication: Variant-rich teaching and decision-focused assessment. Demonstrated action benefit: Some generic simulation or ultrasound benefits exist, but VASC-N transfer and patient benefit are untested.	False certainty, brittle landmark skill, delayed escalation, poor transfer to diverse patients.	May include canonical anatomy plus variability, dynamic assessment, stop or escalation decisions, documentation, and assessment of reasoning as well as hand skills.	Recognition; decision quality; escalation; retention; transfer; equity.

Evidence-status terms are defined in Section 2.5 and Section 3.1. Examples are not exhaustive. Responses are author-proposed, context-dependent options rather than universal recommendations and must remain within local scope of practice, credentialing requirements, available resources, and institutional policy. Most responses are inferred implications; demonstrated action benefit is identified only when directly tested. “Potential failure or harm” does not imply proven causation unless a cited study directly demonstrated an event or association. The integrated VASC-N sequence has not been shown to improve outcomes.

**Table 3:** Proposed operational domains of VASC-N with explicit evidence status and current validation status.

Domain	Core Question	Author-Proposed Observable Behaviours	Illustrative Stop or Escalation Triggers	Evidence Status and Current Validation Status
V—Verify	Why is access required, with what therapy, urgency, duration, and preservation constraints?	Proposed behaviours may include confirming indication, urgency, infusate, anticipated dwell, repeated-sampling need, protected limbs, existing devices, dialysis priorities, and the local pathway.	Inappropriate route/device; unresolved prior complication; protected/altered limb; no clear indication.	Direct finding or recommendation: Access-planning literature and professional guidance [25,26,27,28,51,52]. Inferred implication: Integration of these elements within one VASC-N domain. Demonstrated action benefit: The VASC-N domain has not been validated.
A—Assess	What patient-specific history, anatomy, risk, role, and preference alters the plan?	Proposed behaviours may include reviewing difficult access, thrombosis, surgery, lymph-node procedures, trauma, fistula or graft, previous successful sites, anticoagulation, tissue state, anxiety, and patient knowledge.	History suggests thrombosis/major alteration; patient reports an unsafe route; no acceptable site after basic assessment.	Direct finding: Observational studies and case evidence [21,22,23,24,25,26,27,28,29,30,46,47,48,49,50,58,59,60,61,62,63,64,65,66]. Inferred implication: Integration of history, anatomy, risk, role, and preference. Demonstrated action benefit: The behaviour–outcome link requires testing.
S—Scan and distinguish	What structure is present now, and how certain is its identity and relationship?	A proposed graded sequence may include visual assessment; palpation; dynamic positioning or compression; B-mode ultrasound when indicated; Doppler for flow or identity; real-time guidance for needle control; and post-procedure imaging when route or tip requires confirmation.	Ambiguous identity; non-compressibility; unsafe artery/nerve adjacency; no controlled path; anatomy changes with position.	Direct finding or recommendation: Living and cadaveric mapping plus professional guidance [13,14,15,16,17,18,19,20,21,22,23,24,25,26,27,28,29,30,41,42,43,44,45,46,47,48,49,50,51,52,53,54,55,56,57,58]. Inferred implication: The proposed graded sequence and its placement in VASC-N. Demonstrated action benefit: Context-specific ultrasound benefits do not validate this integrated domain or universal ultrasound use.
C—Choose, adapt, and confirm	Which site, side, device, length, trajectory, operator, and confirmation strategy fit this patient and local scope?	Depending on role and local policy, proposed behaviours may include adapting site, side, gauge, catheter length, angle, position, guidance, attempt limit, operator expertise, and escalation, with required confirmation of position, function, perfusion, and tip or route.	Loss of tip control; radiating pain; suspected arterial entry; resistance; expanding haematoma; no device–vessel fit; unusual trajectory.	Direct finding or recommendation: Guidelines, observational studies, cohorts, and sentinel cases [15,16,24,25,26,27,28,29,30,43,44,45,46,47,48,49,50,51,52,53,54,55,56,57,58,59,60,61,62,63,64,65,66]. Inferred implication: Integration of adaptation, attempt limits, confirmation, and escalation. Demonstrated action benefit: Effectiveness of the integrated sequence is untested.
N—Note, notify, and nurture learning	What must be documented, communicated, taught, audited, and preserved?	Where relevant, proposed behaviours may include recording attempts, anatomical findings, successful strategy, unsuitable sites, confirmation, complications, patient experience, and future recommendations; handing over or updating access plans; and using cases for teaching or quality improvement.	Persistent pain/swelling, dysfunction, perfusion change, recurrent failure, or a finding likely to affect future care.	Direct finding or recommendation: Case literature, haemodialysis and access reviews, and education studies [27,33,59,60,61,62,63,64,65,66,67,68,69]. Inferred implication: Integration of documentation, handover, teaching, and quality improvement. Demonstrated action benefit: Documentation and learning benefits require prospective evaluation.

Evidence-status terms are defined in Section 2.5 and Section 3.1. All behaviours and triggers in Table 3 are author-proposed and context-dependent; they do not constitute universal recommendations. VASC-N does not replace institutional standards, profession-specific regulation, credentialing, supervision, or procedure-specific competency assessment. Neither the individual domains nor the five-domain sequence have been shown to improve patient safety, procedural success, educational performance, or clinical outcomes. VASC-N should initially be used only as a formative conceptual aid until validation supports any higher-stakes interpretation.

**Table 4:** Proposed purposes for publishing patient-specific vascular anatomy in nursing and health professions education journals.

Publication Purpose	Core Question	Suitable Study Designs	Priority Outcomes
Investigate possible harm mechanisms	Which anatomical feature or relationship may contribute to arterial, neural, thrombotic, infiltrative, ischaemic, or route-related harm?	Prospective mapping; incident/near-miss analysis; case-control or cohort study; sentinel case with verified anatomy.	Arterial/nerve injury, haematoma, infiltration, perfusion change, delayed recognition, rescue intervention.
Improve procedural selection	Does anatomy change side, site, device, catheter length, trajectory, position, or operator?	Ultrasound mapping linked to procedural decisions; pragmatic pathway evaluation; decision-impact study.	First-pass/overall success, attempts, time, device appropriateness, catheter-to-vein fit, dwell.
Interpret device behaviour or imaging	Can a variant explain an unexpected catheter course, waveform, blood return, dysfunction, or radiograph?	Diagnostic-accuracy study; multidisciplinary case series; imaging-clinical correlation.	Correct classification, time to confirmation, inappropriate manipulation/removal, adverse events.
Preserve vessels and access longevity	Does recognition protect future peripheral or haemodialysis access?	Longitudinal access-map study; registry; implementation or quality-improvement project.	Access survival, infiltration, thrombosis, abandonment, repeated site loss, dialysis adequacy.
Strengthen nursing education	Can learners recognise the variant, uncertainty signal, stop rule, and escalation pathway?	Simulation, ultrasound curriculum, deliberate-practice or competency study.	Recognition accuracy, decision quality, escalation, retention, transfer to diverse patients.
Advance person-centred care and equity	How do anatomy, body habitus, age, prior procedures, and patient knowledge affect experience and access?	Mixed methods; patient-reported outcome/experience study; stratified imaging cohort.	Pain, anxiety, trust, participation, delay, differential success across patient groups.
Improve documentation and handover	Can patient-specific anatomy be reused rather than rediscovered?	Electronic-record intervention; access passport; audit-feedback study.	Documentation completeness, repeat failures, time to successful access, continuity across encounters.

## Data Availability

The exact database strategies, preliminary minimum reporting checklist, and VASC-N teaching prompts remain in Appendix A, Appendix B and Appendix C.

## Public Involvement Statement

No patients or members of the public were involved in the design, conduct, reporting, or dissemination planning of this review.

## Guidelines and Standards Statement

This targeted narrative review was drafted with reference to SANRA (Scale for the Assessment of Narrative Review Articles) [35].

## Use of Artificial Intelligence

During manuscript preparation and revision, the authors used OpenAI ChatGPT (GPT-5.6 Pro; accessed August 2026) for English-language editing, structural suggestions, and assistance with the layout of conceptual figures and tables. The tool was not used to make autonomous eligibility decisions, generate or alter research data, perform risk-of-bias assessment, or determine the scientific conclusions. The authors independently checked the database records, citations, numerical results, interpretations, tables, figures, and all revised text; they retain full responsibility for the originality, validity, integrity, and final content of the manuscript.

## Abbreviations

The following abbreviations are used in this manuscript:

CCA	Common carotid artery
CVC	Central venous catheter
IJV	Internal jugular vein
PICC	Peripherally inserted central catheter
PLSVC	Persistent left superior vena cava
VASC-N	Vascular Anatomy Safety Competencies for Nursing

## Appendix A. Reproducible Database Search Strategies

### Appendix A.1. PubMed/MEDLINE

( “Anatomic Variation”[Mesh] OR “anatomical variant*”[tiab] OR “anatomic variant*”[tiab] OR “variant anatomy”[tiab] OR “vascular variation*”[tiab] OR “venous variation*”[tiab] OR “arterial variation*”[tiab] ) AND ( “Blood Vessels”[Mesh] OR “Veins”[Mesh] OR “Arteries”[Mesh] OR vascular[tiab] OR vein*[tiab] OR venous[tiab] OR arter*[tiab] ) AND ( “Nursing”[Mesh] OR “Nursing Care”[Mesh] OR nurs*[tiab] OR “Phlebotomy”[Mesh] OR venipuncture[tiab] OR venepuncture[tiab] OR phlebotom*[tiab] OR cannulat*[tiab] OR catheter*[tiab] OR injection*[tiab] OR “Vascular Access Devices”[Mesh] OR “blood sampling”[tiab] OR “arterial puncture”[tiab] OR “central venous access”[tiab] OR PICC[tiab] OR midline[tiab] OR ultrasound[tiab] )

### Appendix A.2. Web of Science Core Collection

TS=( (anatomic* NEAR/2 (variant* OR variation*) OR “variant anatomy”) AND (vascular OR venous OR vein* OR arterial OR arter*) AND (nurs* OR venipuncture OR venepuncture OR phlebotom* OR cannulat* OR catheter* OR injection* OR “vascular access” OR “blood sampling” OR “arterial puncture” OR “arterial line*” OR “central venous access” OR PICC OR midline OR ultrasound) )

## Appendix B. Proposed Minimum Reporting Checklist for Nursing Studies of Vascular Anatomical Variation

The proposed minimum reporting items are presented in Table A1 and should be read alongside the publication purposes in Table 4.

**Table A1:** Minimum reporting checklist for nursing-focused vascular anatomical-variation research.

Item	Minimum Information
1. Anatomical construct	Define the exact vessel, relationship, or continuous/dynamic feature. Distinguish congenital, continuous, dynamic, acquired, and suspected anatomy.
2. Denominator and unit	Report individuals, limbs, sides, vessels, procedures, or images; state laterality and missing data.
3. Ascertainment	Describe dissection, ultrasound/Doppler, CT/MRI, fluoroscopy, surgical observation, or other confirmation; report positioning and physiological conditions.
4. Observer competence	Report training, blinding where applicable, repeated assessment, and inter-/intra-rater reliability for subjective classifications.
5. Nursing task and context	Name the procedure, device, therapy, setting, urgency, operator role, scope, and available escalation resources.
6. Mechanism	Explain how the anatomy could cause misidentification, unsafe adjacency, failed access, malposition, thrombosis, infiltration, dysfunction, pain, or access loss.
7. Recognition cue	Specify the bedside, ultrasound, Doppler, waveform, radiographic, or patient-reported finding that may reveal the problem.
8. Action	Report any action considered or taken—including site, side, position, device, length, trajectory, guidance, attempt limit, confirmation, monitoring, stop rule, or escalation—and state whether its benefit was directly measured, guideline-supported, or author-inferred.
9. Outcomes	Include patient-centred and nurse-sensitive outcomes, not anatomy alone: attempts, pain, complications, time, dwell, patency, experience, preservation, or learning.
10. Transfer and limitations	Describe documentation and handover, educational or policy implications, external validity, uncertainty, and a validation plan; explicitly distinguish direct findings, inferred implications, and demonstrated action benefits; avoid causal claims from descriptive evidence.

This original checklist is a preliminary author-developed proposal for study design and reporting. It has not been formally validated, tested for reliability or feasibility, shown to improve manuscript quality, or designed as a risk-of-bias instrument.

## Appendix C. VASC-N Bedside and Teaching Prompts

The bedside and teaching prompts derived from VASC-N are presented in Table A2 and operationalise the proposed domains summarised in Figure 2 and Table 3. They are formative prompts and have not been validated for high-stakes competency assessment.

**Table A2:** Preliminary VASC-N prompts for bedside preparation, simulation debriefing, and formative competency assessment.

Domain	Prompt
V—Verify	What is the indication? What therapy and dwell are expected? Which limbs or vessels must be preserved? Is this the right device and urgency pathway?
A—Assess	What has happened before? What does the patient know? Are there prior devices, thrombosis, surgery, fistula/graft, oedema, scarring, frailty, or anxiety?
S—Scan and distinguish	Is the target unquestionably a patent vein or intended artery? What are its depth, diameter, course, compressibility, and relation to nerves/arteries? Does position change it?
C—Choose, adapt, and confirm	Which side, site, device, length, angle, position, guidance method, operator, and attempt limit could fit this patient and local scope? Which confirmation is required by the procedure and local policy?
N—Note, notify, and nurture	What information may be relevant to the next clinician and the patient? What failed or succeeded? Which sites may be unsuitable? Could this case require handover, an access plan, teaching, audit, or policy review?
